# Variation and diversification of the microbiome of *Schlechtendalia chinensis* on two alternate host plants

**DOI:** 10.1371/journal.pone.0200049

**Published:** 2018-11-08

**Authors:** Hai-Xia Wu, Xiaoming Chen, Hang Chen, Qin Lu, Zixiang Yang, Weibin Ren, Juan Liu, Shuxia Shao, Chao Wang, Kirst King-Jones, Ming-Shun Chen

**Affiliations:** 1 Research Institute of Resource Insects, Chinese Academy of Forestry, Kunming, China; 2 The Key Laboratory of Cultivating and Utilization of Resources Insects, State Forestry Administration, Kunming, China; 3 Southwest Forestry University, Bailongsi, Kunming City, Yunnan, PR. China; 4 Department of Biological Sciences, University of Alberta, Biological Sciences Bldg., Edmonton, Alberta, Canada; 5 Department of Entomology, Kansas State University, Manhattan, KS, United States of America; Oklahoma State University, UNITED STATES

## Abstract

*Schlechtendalia chinensis*, a gall-inducing aphid, has two host plants in its life cycle. Its wintering host is a moss (typically *Plagiomnium maximoviczii*) and its main host is *Rhus chinensis* (Sumac), on which it forms galls during the summer. This study investigated bacteria associated with *S*. *chinensis* living on the two different host plants by sequencing 16S rRNAs. A total of 183 Operational Taxonomic Units (OTUs) from 50 genera were identified from aphids living on moss, whereas 182 OTUs from 49 genera were found from aphids living in Sumac galls. The most abundant bacterial genus among identified OTUs from aphids feeding on both hosts was *Buchnera*. Despite similar numbers of OTUs, the composition of bacterial taxa showed significant differences between aphids living on moss and those living on *R*. *chinensis*. Specifically, there were 12 OTUs from 5 genera (family) unique to aphids living on moss, and 11 OTUs from 4 genera (family) unique to aphids feeding in galls on *R*. *chinensis*. Principal Coordinate Analysis (PCoA) also revealed that bacteria from moss-residing aphids clustered differently from aphids collected from galls. Our results provide a foundation for future analyses on the roles of symbiotic bacteria in plant-aphid interactions in general, and how gall-specific symbionts differ in this respect.

## Introduction

Insects harbour a wide range of symbiotic microbes, but the actual species composition can vary between different developmental stages [1, 2]. In general, bacterial symbionts are comprised of two kinds: obligate symbionts and facultative symbionts. Obligate endosymbionts are necessary for insect growth and development, and usually supply important nutrients such as essential amino acids to the host insect [3, 4]. In contrast, facultative symbionts are not essential for growth and development of their host, but may improve fitness. For instance, some facultative symbionts boost the defense system of the host and thus enhance resistance to natural enemies and pathogens [5–7]. Typically, facultative symbionts have not shared a long evolutionary relationship with their host insects [8].

Aphids are a large group of insects belonging to the Aphidiodea superfamily. Many aphid species are destructive pests of crops and forests. Symbiosis with microorganisms is critical for the development of aphids. One of the essential symbiotic bacteria associated with aphids is *Buchnera aphidicola*, an obligate endosymbiont that provides essential amino acids, which are either absent or scarce in plant sap [9]. In addition to its obligate symbionts, aphids can also harbor several facultative bacterial symbionts, which can be mutualistic in the context of various ecological interactions. *Serratia symbiotica* is one of the most common facultative symbiotic bacteria in aphids [10]. *S*. *symbiotica*, together with other symbionts, provides aphids with protection against parasitoids [11]. Because of different ecological habitats, different aphid species harbor specific bacterial taxa for specific functions [12].

The aphid *Schlechtendalia chinensis*, a member of the subfamily Pemphiginae, is a beneficial species with great economic and medicinal values [13, 14]. The aphid induces the formation of large, single-chambered galls on its host plant, and some chemicals of the galls are used as ingredient traditional Chinese medicine to treat various conditions including cough, diarrhea, night sweats, dysentery, and internal bleeding [15]. In the mining industry, some applications use chemicals from *S*. *chinensis*-induced galls to extract rare metals. In addition to its economic and medicinal values, *S*. *chinensis* also has a fascinating biology. The aphid has a life cycle of sexual and asexual reproduction and a switch of host plant species during its life cycle. The primary host of *S*. *chinensis* is *Rhus chinensis* (Sumac tree) and the secondary host is *Plagiomnium maximoviczii* (moss). In early spring in China, sexuparae migrate from mosses to the trunk of Sumac trees to produce sexually reproductive females and males that subsequently mate to produce fundatrix, which feed on rachides wings and induce the formation of galls [16, 17]. Within a gall, *S*. *chinensis* undergoes cyclical parthenogenesis for multiple generations to increase population sizes rapidly. In late fall, the galls break up and the alate fundatrigeniae of *S*. *chinensis* emerge from galls and relocate their secondary host mosses, where aphids over-winter.

Based on its unique biology and its ability to induce galls, we hypothesized that *S*. *chinensis* harbors a set of unique symbiontic taxa, which are likely to play critical roles in its biology, such as mediating adaptive responses on different hosts, or contributing to its gall-inducing ability. The rapid advance in high throughput sequencing technologies has made it possible to comprehensively investigate the compositions of microbial community in small insects [18]. In this study, we examined the diversity of bacterial communities associated with *S*. *chinensis* in different hosts via next-generation sequencing. Specifically, we sequenced the variable regions 4–5 of 16S rRNA amplicons using the Illumina sequencing platform (using barcode Illumina paired-end sequencing, BIPES) to characterize the composition and diversity of microbiomes during different developmental stages of *S*. *chinensis*.

The experimental protocol was approved by the Research Ethics Committee of Research Institute of Resource Insects, Chinese Academy of Forestry and the written informed consent were obtained from all subjects. The Research Institute of Resource Insects, Chinese Academy of Forestry issued the permission for each location. We confirm that the field studies did not involve endangered or protected species.

## Materials and methods

### Aphid collection

We collected aphid samples corresponding to nine time points (one per month) directly from host plants from March to November, 2015, in Kunming, Yunnan province, China. Each condition was represented by three independent replicates. In order to observe potential dynamic changes in the diversity of microbal taxa, samples were collected every month, and for consistency, we chose the middle of each month. The April samples represented the fundatrix stage. Samples from March, April, and November were taken as the group of insects from the host moss, whereas the remaining samples were taken from Sumac trees.

To obtain insects from galls, individual galls were soaked in 75% ethanol by gently shaking for 30 s. The gall was washed three times with doubly distilled (dd) water. Using sterile conditions, an incision into the gall was carried out with a blade. Aphids were taken out individually with forceps and transferred into individual tubes. After removing any environmental contaminants, the insect samples were frozen at −80°C for subsequent genomic DNA extraction. For moss-residing aphids, 30 individual aphids were collected and transferred into a microfuge tube. Aphids were washed with 1 ml of 75% ethanol by gently shaking for 60 s. The insects were then stored in −80°C for DNA extraction.

### Extraction of genome DNA

Total genomic DNA from a sample was extracted using a DNA Kit following the directions provided by the manufacturer. DNA concentration and purity was monitored on a 1% agarose gel. DNA concentration was diluted to 1 ng/μl with sterile water and used as template for PCR amplification.

### Amplicon generation

The variable regions 4–5 (16SV4-V5) in 16S rRNA genes were amplified using a pair of specific primers, namely 515F “5- GTGCCAGCMGCCGCGG-3” and 907R”5- CCGTCAATTCMTTTRAGTTT-3”, with barcodes. All PCR reactions were carried out with PCR Master Mix. A PCR reaction mix contained 5 μl 5x reaction buffer, 5 μl 5x GC buffer, 2 μl dNTP (2.5 mM),1 μl Forward primer and Reverse primer, respectively (10 mM), 2 μl DNA Template, 8.75 μl ddH2O, and 0.25 μl Q5 DNA Polymerase. PCR amplification was achieved by an initial denaturation at 98°C for 2min, followed by 25–30 cycles of denaturation (98°C for 15s), annealing (55°C for 30s), and extension (72°C for 30s). A final extension step at 72°C for 5 min was included at the end of PCR amplification.

### PCR amplification and sequencing

PCR products were mixed with the same volume of 1x loading buffer and analyzed on a 2% agarose gel. Samples with a main 400-450bp band were purified with a Gel Extraction Kit. Amplicons were then quantified and sequencing libraries were generated using Next Ultra DNA library Prep Kit for Illumina according to manufacturer’s recommendations. The amplicon libraries were subsequently sequenced on an Illumina MiSeq platform at company.

The sequencing data were submitted to NCBI (National Center for Biotechnology Information) GenBank (accession numbers: SRP159626).

### Bioinformatics analyses

Paired-end reads were merged using FLASH [19]. Low quality reads were removed. Sequences were analyzed with the QIIME software package [20] using default parameters for each step. The Uparse software [21] was used to cluster the sequences into operational taxonomic units (OTUs) at an identity threshold of 97%. The RDP Classifier [22] was used to assign each OTU to a taxonomic level. Other analyses, including rarefaction curves, Shannon index, and Good’s coverage, were performed with QIIME. In addition, the OTU table produced by the QIIME pipeline was imported into MEGAN 4 and mapped on the GreenGene Database [23, 24].

Principal Coordinate Analysis (PCoA) was performed to get principal coordinates and visualize from complex, multidimensional data. PCoA has been recognized as a simple and straight-forward method to group and separate samples in a dataset. In this study, PCoA was used to analyze the sequencing results using WGCNA package, stat packages and ggplot2 package in R software.

### Statistical methods

All experimental results were evaluated using analysis of variance (ANOVA) for multiple comparisons followed by the Turkey test. Differences were considered significant at p < 0.05. All analyses were done using the programs WGCNA, STAT, and ggplot2 in the R software package.

## Results

### Sequence reads and OTUs

In total, 975,583 raw reads were obtained from all samples, and after filtering, 973,373 high-quality sequences were retained and subsequently clustered into 194 OTUs (S1 Table). Of the 194 OTUs, five were classified as Archaea, 83 were Bacteria, and the remaining 106 remain to be determined since they showed no blast hits. Of the 83 bacterial OTUs, 56 (67.5%) were Proteobacteria, 10 belong to Firmicutes, and the remaining 17 belong to Cyanobacteria and others.

The distribution of reads and OTUs during different seasons is shown in Table 1, along with other parameters including Chao1 Index, Shannon Index, and Good's coverage. The highest number of reads was obtained in the sample collected on October 19, but the highest number of OTUs was obtained in the sample collected on March 24. In comparison, the lowest number of reads was obtained in the sample collected on May 27, whereas the lowest number of OTUs was obtained in the sample collected on September 16.

**Table 1:** Distribution of OTUs among samples collected on different time.

Sample date	11/30	3/24	4/14	5/27	6/10	7/09	8/12	9/16	10/19
**Clean reads**	98,653	104,744	111,689	86,722	117,146	117,901	100,097	117,142	119,279
**OTU**	98	146	131	128	82	145	119	64	87
**Chao1 Index**	113.1667	164.0714	156.0714	143	94.83333	153	139.7143	79	96.1
**Shannon Index**	0.549917	0.267498	0.198749	0.449417	0.246772	0.711832	0.154277	0.103239	0.108371
**Good’s coverage**	0.99986	0.99977	0.99973	0.9997	0.99978	0.99984	0.9997	0.99985	0.99986

### Major and minor OTUs

The 194 OTUs were divided into six groups based on their highest number of sequence reads at any time point (Table 2). Group 1 contains only one OTU, which is highly abundant with sequence reads over 30,000 in all samples at different time points. This OTU belongs to *Buchnera*, strongly suggesting that this is the principal endosymbiont of *Schlechtendalia chinensis*. Group 2, which comprises also highly abundant taxa, contains six OTUs with the highest numbers of reads between 100 to 2,000 per sample. The six OTUs belong to different taxonomic units including *Bacillus*, *Limnohabitans*, *Candidatus Cloacamonas*, *Comamonadaceae* and *Pseudomonas*. Group 3, which defined here as medium-abundant taxa, contains 38 OTUs with the highest numbers of reads between 10 to 100 in different samples. Twenty-five OTUs had no blast hit, whereas the remaining OTUs corresponded to different taxa and are listed in the Table 2. Group 4, which represents low- abundant taxa, contains 47 OTUs with the highest read numbers between 5 and 10 in different samples. Twenty-eight OTUs had no blast hit, whereas the remaining OTUs corresponded to different taxonomic units. Group 5, comprises infrequenttaxa, contains 79 OTUs with the highest numbers of reads only between 2 to 5 in different samples, whereas group 6, referred as rare taxa, contains 22 OTUs with the highest number of reads below 2.

**Table 2:** Groups of associated microbes with different levels of sequence reads. The lowest known level of taxonomy is given in the table. The numbers in parenthesis are the numbers of OTUs in that taxonomic unit.

Group	Sequence reads	OTUs	OTU taxonomy
**1**. Super abundant OTU	>30,000	1	Buchnera (1)
**2**. Highly abundant OTUs	<2,000–100	6	Bacillus (1), Limnohabitans (1), Candidatus Cloacamonas (1), Comamonadaceae (1) Pseudomonas (2)
**3**. Medium Abundant OTUs	<100–10	38	Comamonas (1), Lactobacillales (1), Buchnera (2), Anaerolinaceae (1), Stenotrophomonas (1), Pirellulaceae (1), Rhodococcus (1), Streptococcus (1), Acinetobacter (1), Thermus (1), Crenarchaeota (1), Comamonadaceae (1), no blast hit (25)
**4**. Low abundant OTUs	<10–5	47	Xanthomonadaceae (1), Bacillus (2), Bacteria (1), Gemmataceae (1), Rhodobacter (1), Aminobacterium (1), Nitrosopumilus (1), Sphingomonas(1), Archaea (2), Pseudoxanthomonas (1), Buchnera (1),Anaerolinaceae (1), Methyloversatilis (1), Propionibacterium (1), Leuconostoc (1), Oxalobacteraceae (1), Anaerolinaceae (1), no blast hit (28)
**5**. Infrequent OTUs	<5–2	79	Buchnera (7), Alteromonadales (1), Comamonadaceae (7), Phormidium (1), Allochromatium (1), Rhizobium (1), Staphylococcus (1), Anoxybacillus kestanbolensis (1), Mycoplana (1), Archaea (1), Xenococcaceae (1), Enterobacteriaceae (3), Streptophyta (1), Bacillales (1), Pseudomonas stutzeri (1), Streptophyta (2), Acinetobacter (1), Halomonas (1), Methylobacteriaceae (1), Brevibacillus reuszeri (1), Roseomonas rosea(1), Caulobacteraceae (1), Burkholderiales (1), Rhizobiales (1), Wolbachia (1), no blast hit (39)
**6**. Rare OUTs	<2–1	22	Buchnera (5), Thauera (1), Brevundimonas diminuta (1), Enhydrobacter (1), no blast hit (14)

### OTUs associated that differ between host plants

**Fig 1:**
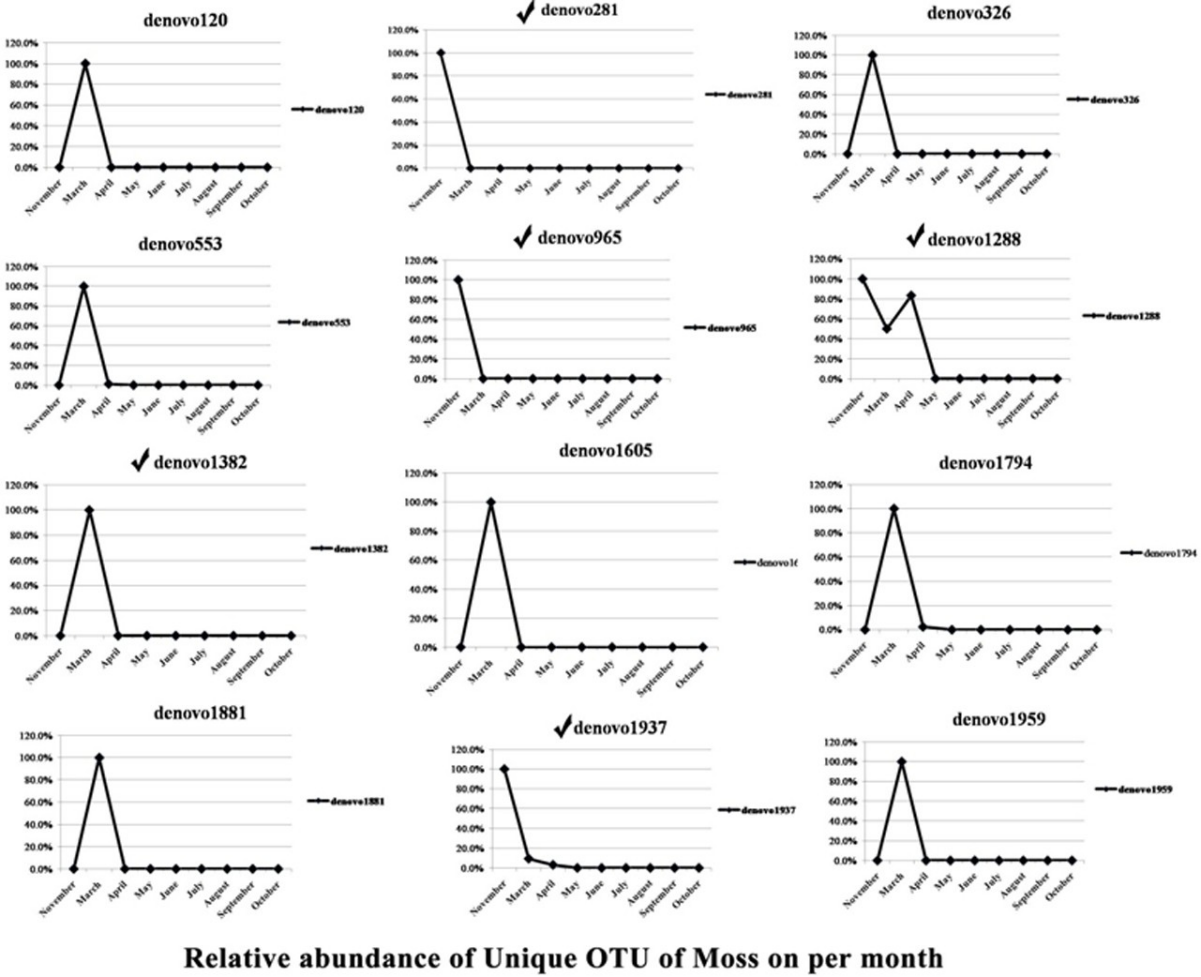
Dynamic changes at different sampling times of OTUs specific to aphids feeding on moss (from Nov to Apr). The symbol ✓ represents these OTUs that were classified into genus or family. The remaining OTUs had no blast hits.

**Fig 2:**
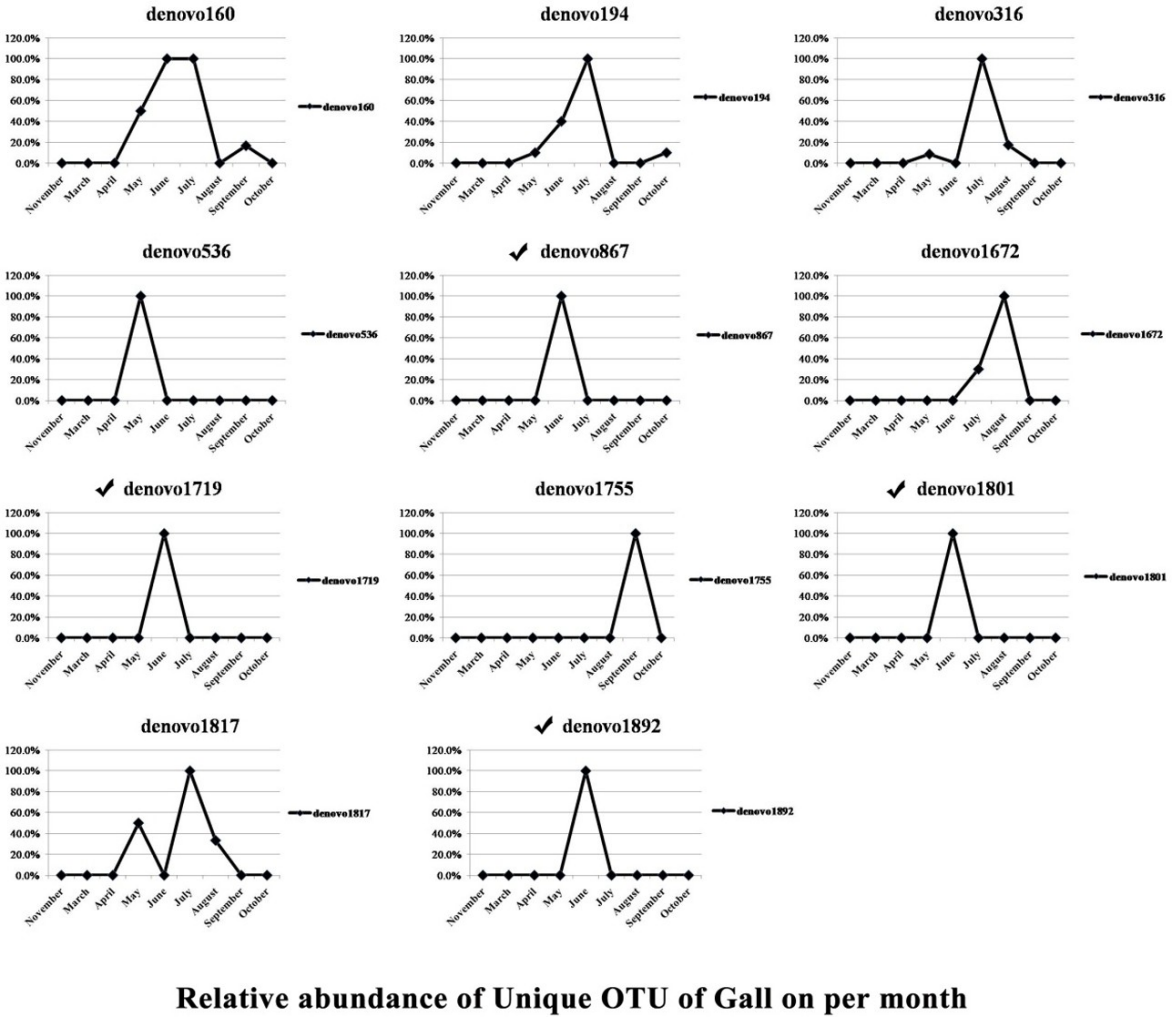
Dynamic changes at different sampling times of OTUs specific to aphids feeding in galls on Sumac trees (May to Oct when aphids lived in galls). The symbol ✓ indicates the OTUs that were classified into genus or family. The remaining OTUs had no blast hits.

Samples collected in March, April, and November were isolated from the host moss, whereas the remaining samples were obtained from Sumac galls. There were 12 OTUs found exclusively in aphids collected from moss, and 11 OTUs were present exclusively in aphids collected from Sumac galls. The dynamic changes at different sampling time points of these OTUs specific to aphids feeding on the two different hosts are shown in Figs 1 and 2. The OTUs specific to aphids on different host plants were also listed in Table 3. There are five genera that were detected only in moss-residing aphids. These, included *Wolbachia*, a genus from the family Gemmataceae, a genus from the family Pirellulaceae, *Phormidium*, and *Streptococcus*. And there are four genera (family) that were detected only in aphids feeding in Sumac galls, including *pGrfC26*, a genus from the family Xenococcaceae, a genus from the phylum Crenarchaeota, and *Nitrosopumilus*.

**Table 3:** OTUs associated with aphids feeding on two different host plants.

Host Plant	OTUs	Taxonomy (OTU name)						
Exclusively in aphids from moss	12	No blast hit (denovo120)	*Wolbachia* (denovo1288)		Gemmataceae (denovo965)	No blast hit (denovo553)	Pirellulaceae (denovo281)	Comamonadaceae (denovo1605)
		*Phormidium* (denovo1382)	No blast hit (denovo1794)		No blast hit (denovo1959)	No blast hit (denovo1881)	No blast hit (denovo326)	*Streptococcus* (denovo1937)
Exclusively in aphids from Sumac	11	*pGrfC26* (denovo1719)	Xenococcaceae (denovo1801)		*Buchnera* (denovo160)	No blast hit (denovo1817)	No blast hit (denovo1672)	No blast hit (denovo316)
		No blast hit (denovo536)	*Crenarchaeota* (denovo867)		*Nitrosopumilus* (denovo1892)	*Streptophyta* (denovo194)	No blast hit (denovo1755)	
Present in aphids from all samples	23	*Buchnera* (denovo528)	*Stenotrophomonas* (denovo2048)		*Buchnera* (denovo915)	*Acinetobacter* (denovo1435)	*Bacillus* (denovo187)	No blast hit (denovo780)
		No blast hit (denovo2067)	*Pseudomonas stutzeri*(denovo739)		*Buchnera* (denovo2027)	*Pseudomonas* (denovo1118)	*Buchnera* (denovo1712)	*Buchnera* (denovo1556)
		*Buchnera* (denovo753)	*Buchnera* (denovo496)		No blast hit (denovo1629)	*Streptophyta* (denovo743)	Buchnera (denovo155)	*Anoxybacillus kestanbolensis* (denovo1177)
		Rhodococcus (denovo1058)	Buchnera (denovo104)		Comamonadaceae (denovo544)	Buchnera (denovo1489)	No blast hit (denovo1464)	

The OTUs were roughly equally present in aphids feeding on the two different hosts (Table 3). There are nine OTUs from different genera or families that were always present in aphids collected at different time points, including *Buchnera*, *Stenotrophomonas*, *Acinetobacter*, *Bacillus*, *Pseudomonas*, *Streptophyta*, *Anoxybacillus kestanbolensis*, Rhodococcus, and Comamonadaceae. The remaining OTUs were detected in aphids residing on either host plant, but their presence was not consistent at all time points.

### Beta diversity of microbiota during the whole life cycle of *S*. *chinensis*

**Fig 3:**
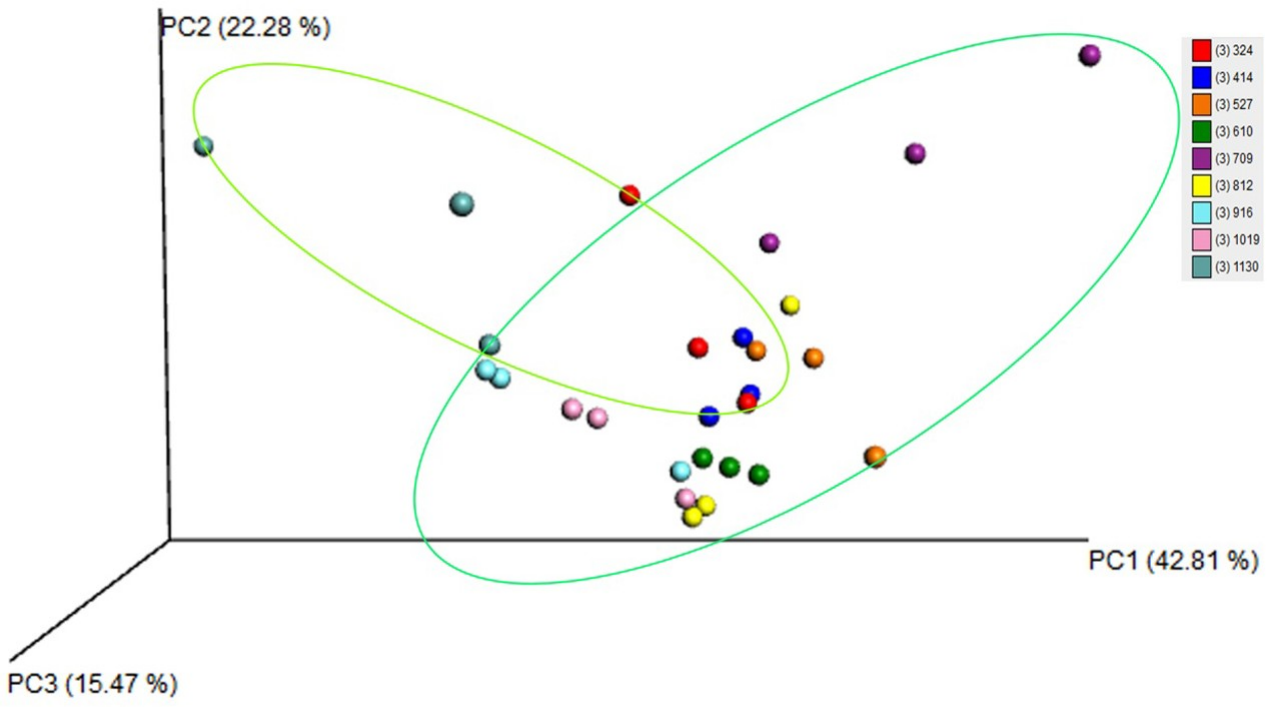
Weighted UniFrac principal coordinates analysis (PCoA) plots based on weighted UniFrac metric. Light green circle indicates data from aphids feeding on the moss. Dark green circle indicates data from aphids feeding in galls on Sumac trees.

A weighted UniFrac principal coordinates analysis (PCoA) was performed to compare the overall structure of microbiota from all samples based on the relative abundance of OTUs (Fig 3). There was an obvious separation between samples even though some samples unevenly distributed presence. PC1, PC2 and PC3 accounted for 42.81%, 22.28% and 15.47% of total variations, respectively. This analysis showed differences in beta diversity resulted in separation between aphids feeding on moss and Sumac galls.

## Discussion

The most abundant OTU was denovo496, which accounted for 80–99% of total sequence reads in all samples (S1 Table, S1 Fig). The taxonomy of denovo496 is *Buchnera*, which is an endosymbiont in all aphid species [3, 25–30]. Since *Buchnera* is an obligate endosymbiont and is present in every aphid cell, it is not surprising that denovo496 was the most abundant microbe detected in our study. The abundance of sequence reads for *Buchnera* suggests that our sequence data were reliable and representative. Interestingly, there were 17 other OTUs corresponding to *Buchnera* in addition to denovo496 (S1 Table). The 17 OTUs were much less abundant with average sequence reads equal to or below 16. However, many of these minor *Buchnera* OTUs, for example the OTU denovo915 (S1 Fig), were consistently distributed in different samples, suggesting that these minor OTUs were not caused by sequence errors or other artifacts. The exact sources for these minor *Buchnera* OTUs remain to be determined. The minor OTUs could be due to minor alleles among *Buchnera* individuals in aphid cells.

The second-most abundant OTU was denovo743, which we detected in all samples across different time points. The percentages of denovo743 reads were below 0.8% of total reads in aphids feeding on moss, but the percentages reached as high as 7.5% of total reads in aphids living in galls. The taxonomy of denovo743 is *Streptophyta* from chloroplasts. Based on its taxonomy and abundance distribution, the sequences corresponding to denovo743 were likely due to contamination of chloroplast DNA. It could be due to chloroplasts from gall tissues that stuck to the outside of aphid bodies. Alternatively, chloroplasts from gall tissues were sucked up to aphid guts.

Aside from *Buchnera*, other major bacteria associated with *S*. *chinensis* were from the genera *Bacillus*, *Limnohabitans*, *Candidatus Cloacamonas*, a genus from the family Comamonadacease, and *Pseudomonas* (Table 2, S1 Table). *Bacillus* is found commonly in aphids [31] and in other insect species [2, 32, 33, 34]. *Bacillus* protects the host from other bacterial and fungal colonization by producing antimicrobial compounds such as phenols [33, 35]. *Pseudomonas* bacteria colonize a wide range of ecological niches [27, 36], but it is not clear what kind of benefits this bacterium provides to aphids.

We detected 23 OTUs in all aphid samples collected from either moss or Sumac galls. Even though they were common to all samples, the abundance of these OTUs varied greatly from sample to sample except for *Buchnera*. Therefore, it is not clear if these common OTUs are obligate or facultative and further research is needed to reveal their impact on the aphid host or aphid-plant interactions.

There were 12 OTUs unique to moss-residing aphid populations (Table 3). Typically, each OTU was detected at only one time point, either in November or March except the OTU denovo1288, which was found in Nov, Mar, and Apr samples (Fig 1). The presence of a specific symbiont at a specific time point indicated that these symbionts are likely facultative and play transient roles in the aphid life cycle. *Wolbachia* was among these moss-specific OTUs. *Wolbachia* has been previously found in aphids and it is a facultative symbiont that plays a role in the reproduction of its host insects [37, 38], as well as a role in host plant fitness [38]. Other symbionts specific to moss-residing aphids included bacteria from the families or genera Comamonadaceae, *Phormidium*, Pirellulaceae, Gemmataceae, and *Streptococcus*.

There were 11 OTUs specific to aphids associated with Sumac galls (Table 3). Most of these were present (or mainly present) in samples from a single time point (Fig 2), suggesting that they are likely facultative in their interaction with the host aphid. *Crenarchaeota* was among the symbionts unique in aphids collected from Sumac galls. *Crenarchaeota* symbionts have been reported to be involved nitrogen cycling [39]. *Nitrosopumilus* was another symbiont specific to aphids feeding in Sumac galls. *Nitrosopumilus* has been found to play roles in ammoxidation and CO2 assimilation in insects [40, 41].

In summary, a comprehensive survey was conducted on microbes associated with *S*. *chinensis* aphids on two different hosts at different time points. This survey identified a range of OTUs from highly abundant to rare microbes from different samples. We discovered both common and plant-specific symbionts. Identification of these symbionts provides a foundation for future studies to determine the roles of different symbionts on aphid physiology and insect-plant interactions.

## Supporting information

S1 Fig Abundance and variation of sequence reads for the two OTUs, denovo496 and denovo915, in different samples collected at different time points. The taxonomy of both OTUs correspond to Buchnera, an obligate endosymbiont. Standard errors are shown on the top of each bar.
(TIF)

S1 Table All OTUs data. (XLS)
