# Supplementary figures and images for: Variation and diversification of the microbiome of Schlechtendalia chinensis on two alternate host plants
Source: PLoS One. 2018 Nov 8;13(11):e0200049. doi: 10.1371/journal.pone.0200049 (PMC6224032; doi:10.1371/journal.pone.0200049)

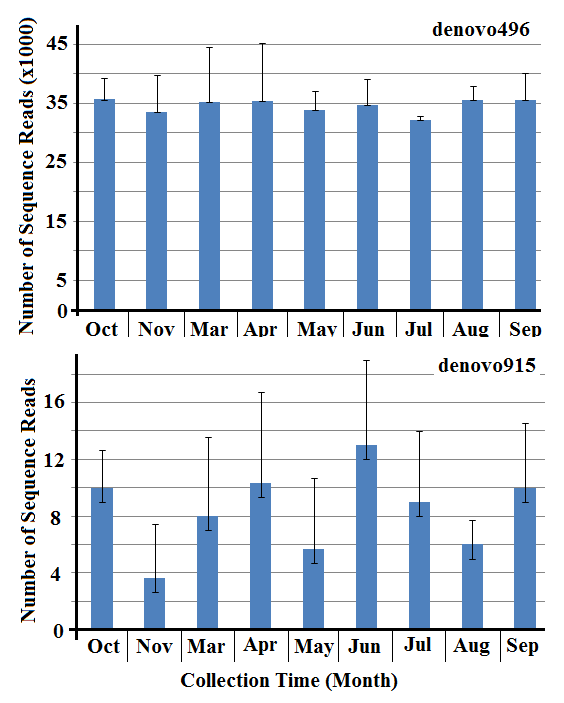

Supplement: S1 Fig — The taxonomy of both OTUs correspond to Buchnera, an obligate endosymbiont. Standard errors are shown on the top of each bar. (TIF) [file pone.0200049.s001.tif]
